# Supplementary material for: A natural timeless polymorphism allowing circadian clock synchronization in “white nights”
Source: Nat Commun. 2022 Mar 31;13:1724. doi: 10.1038/s41467-022-29293-6 (PMC8971440; doi:10.1038/s41467-022-29293-6)
Supplement: Supplementary file 1 — Supplementary Information [file 41467_2022_29293_MOESM1_ESM.pdf]

Supplementary Figure 1

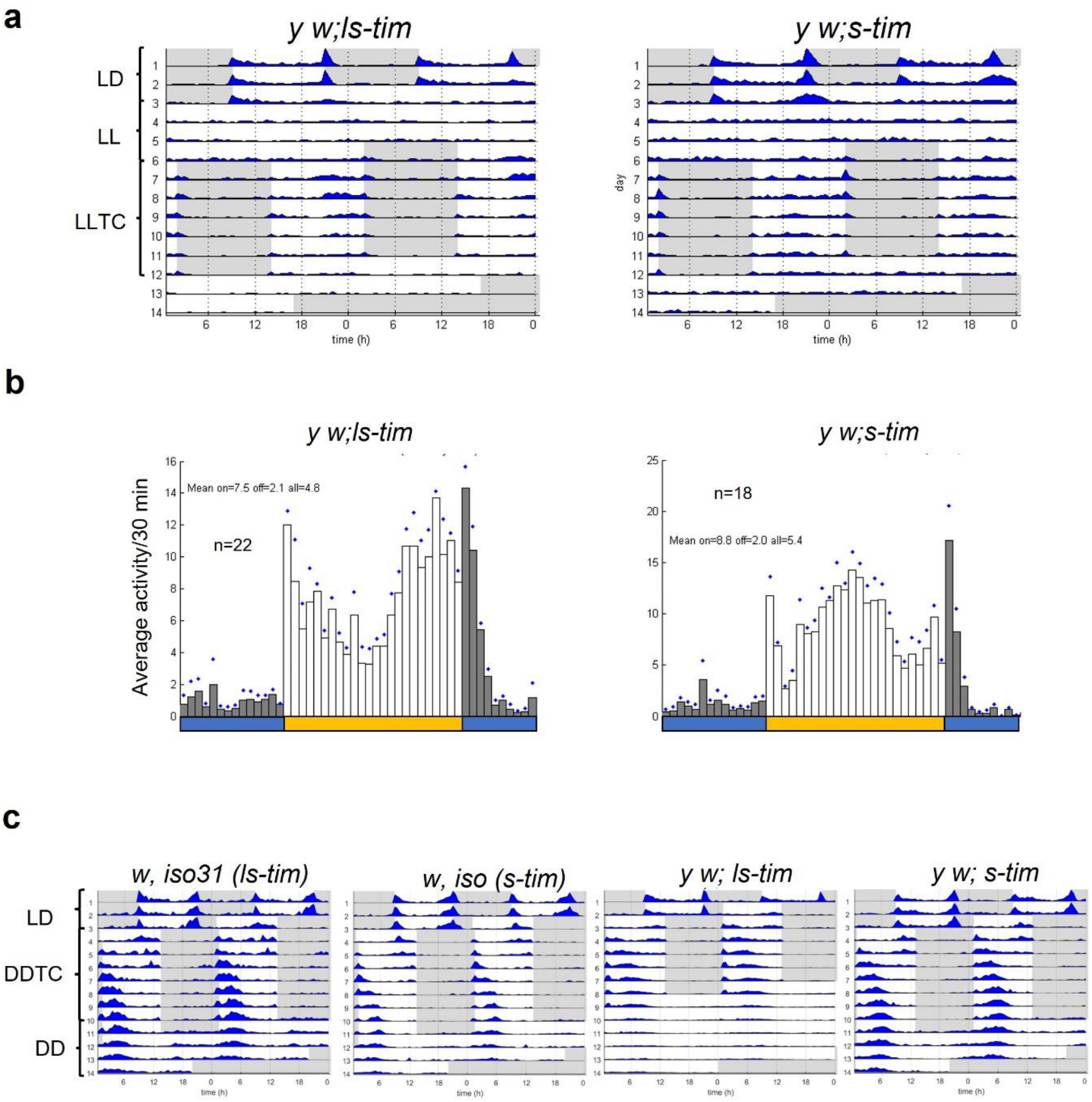

Supplementary Figure 1: *s-tim* flies cannot synchronize their behaviour to temperature cycles in constant light. a) Average actograms of one representative experiment as described in the legend for Fig. 1a. N (*y w; ls-tim*): 22, (*y w; s-tim*): 18. **b)** Histograms of the average activity of days four to six in LLTC. Same flies as in (a).Yellow bar: thermophase, blue bar cryophase (12h each). Blue diamonds indicate SEM. **c)** Average actograms in LD followed by DDTC and DD constant temperature. White areas: lights-on and 25°C during LD, and lights-off

and 25°C during DDTC and DD. Grey areas: lights-off and 25°C during LD, and lights-off and 16°C during DDTC. N (*w, iso31 ls-tim*): 15; (*w, iso s-tim*): 15; (*y w; ls-tim*): 15; (*y w; s-tim*): 17.

Supplementary Figure 2

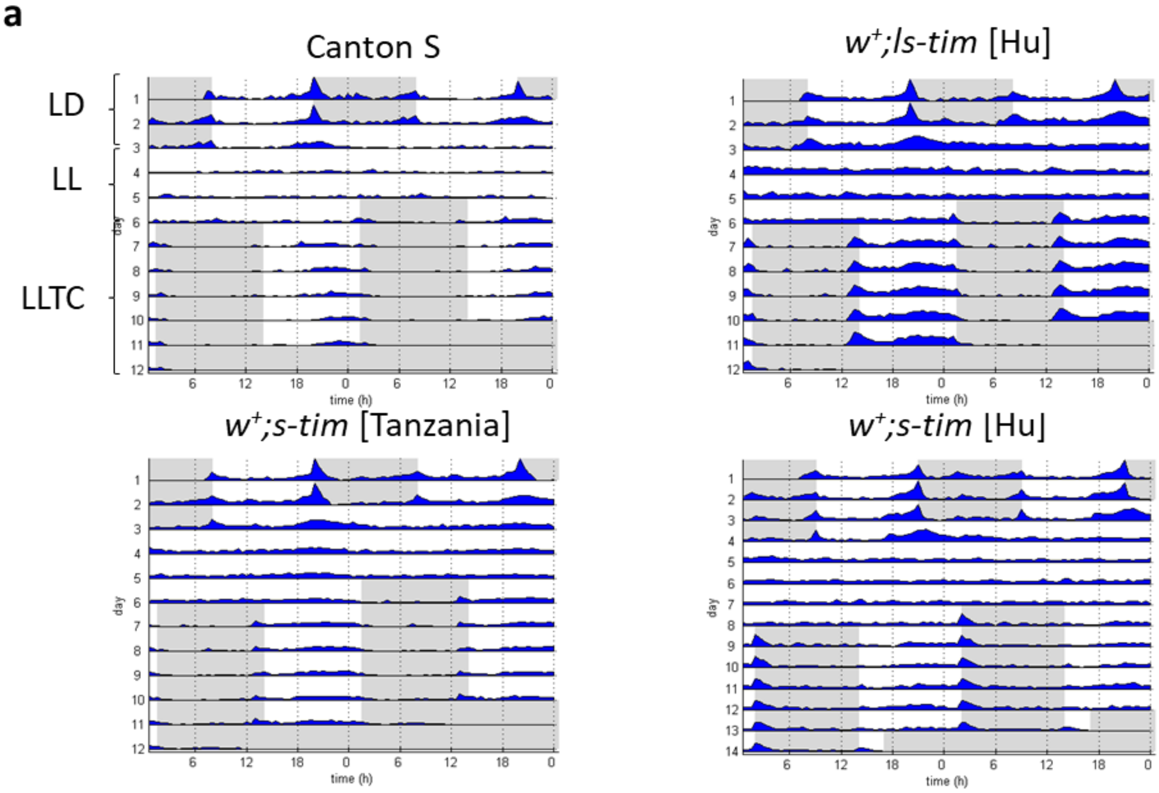

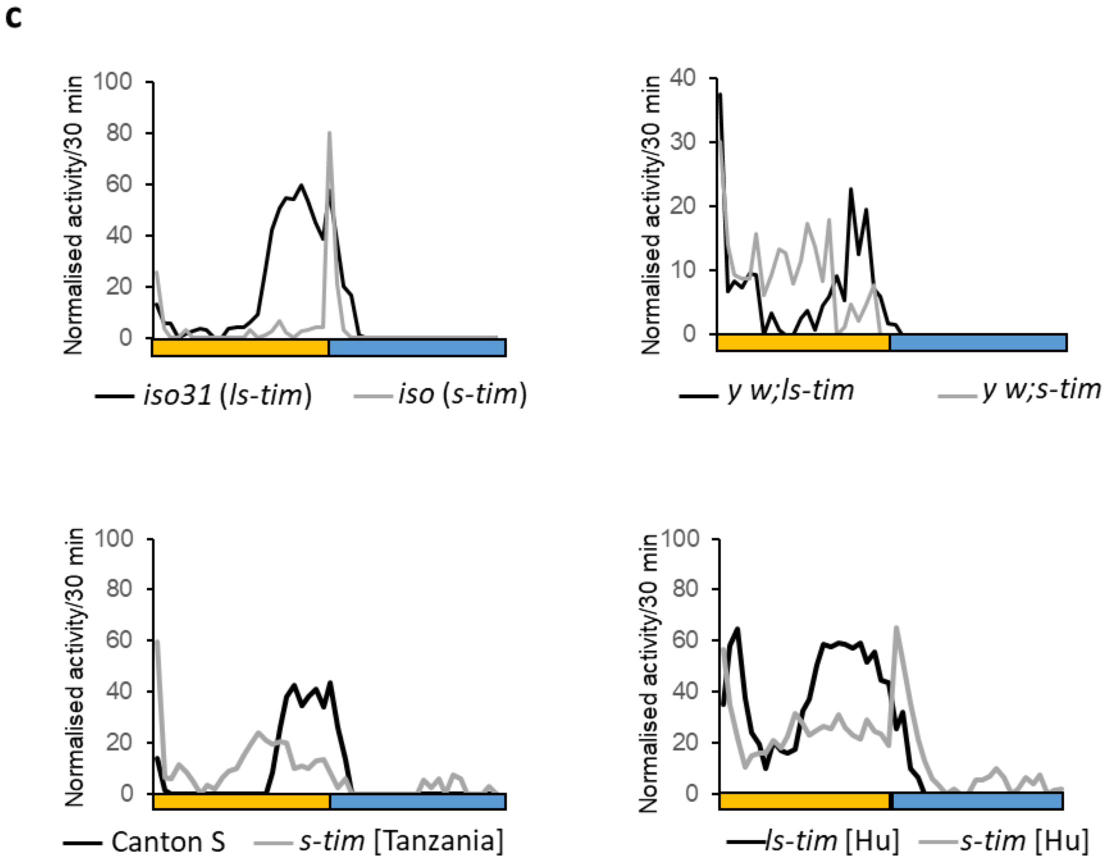

Supplementary Figure 2: *s-tim* in various wild type strains does not support synchronisation to temperature cycles in constant light. a) Group actograms of one representative experiment as described in the legend for Fig. 1a. N (Canton S): 20; (w^{+}*; ls-tim* [Hu]): 24; (w^{+}*; s-tim* [Tanzania]): 19; (w^{+}*; s-tim* [Hu]): 24. **b)** Median of normalised activity during day 6 of LLTC of independent experiments combined. Yellow bar: thermophase, blue bar cryophase (12h each). *iso31 ls-tim* and *iso s-tim* same flies as in Fig. 1c, N (*y w; ls-tim*): 50, (*y w;s-tim*): 52; N (Canton S): 51; (w^{+}*; ls-tim* [Hu]): 43; (w^{+}*; s-tim* [Tanzania]): 19; (w^{+}*; s-tim* [Hu]): 63. Source data are provided as a Source Data file.

Supplementary Figure 3

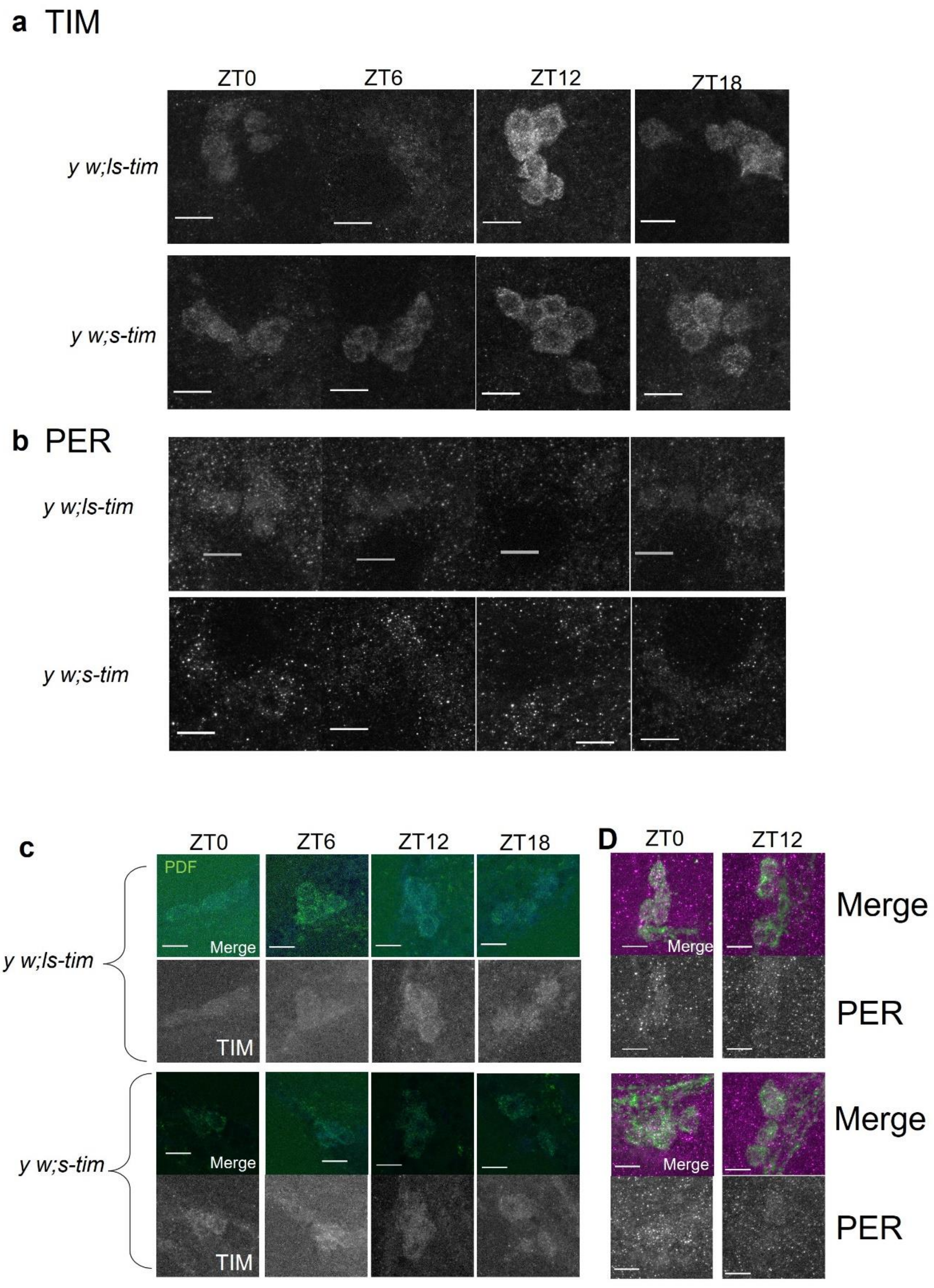

Supplementary Figure 3: Constitutive cytoplasmic localisation of TIM and low PER levels in *s-tim* flies during constant light and temperature cycles. Representative images for the

quantifications shown in Fig. 2. **a, b)** TIM (a) and PER (b) in the LNd on day six of LLTC in *y w; ls-tim* and *y w;s-tim* flies (for quantification see Fig. 2). **c, d)** TIM (c, blue) and PER (d, magenta) in the s-LNv. PDF antibody (green) was used as a marker to identify the LN PDF^+ neurons. Scale bar: 10µm.

Supplementary Figure 4

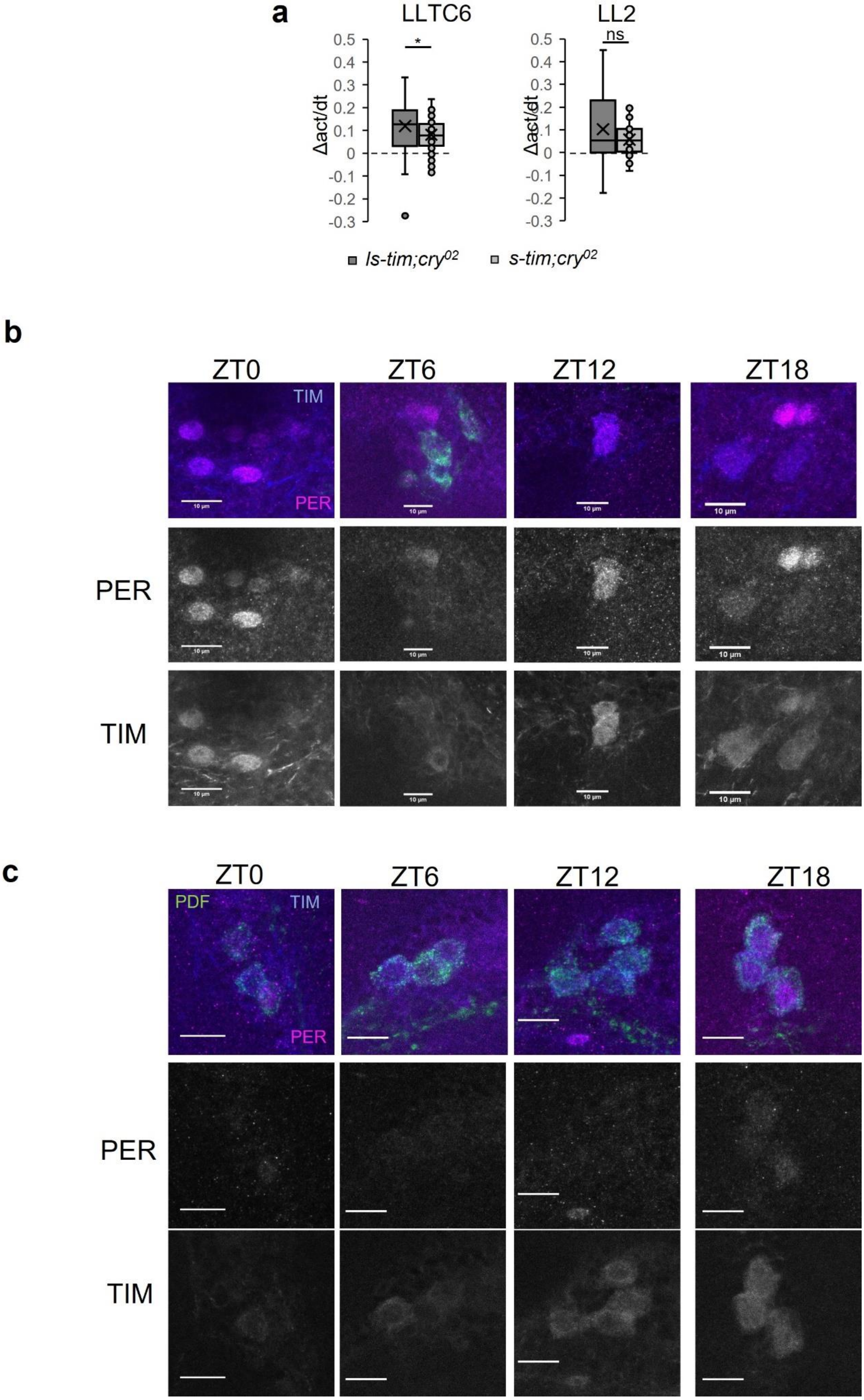

Supplementary Figure 4: Cryptochrome depletion partially restores rhythmic behaviour and molecular oscillations during constant light and temperature cycles in *s-tim* flies. a) Box plots showing the slope of the evening peak on the 6th day of LLTC and the second day of LL after LLTC entrainment. Same flies as in Fig. 3b. In LLTC6 ZT_{min}(*ls-tim;cry*^{02}) = 4, ZT_{min}(*s-tim;cry*^{02}) = 4.5; ZT_{max}(*ls-tim;cry*^{02}) = 9, ZT_{max}(*s-tim;cry*^{02}) = 11.5. In LL2 ZT_{min} = 3 ; ZT_{max}(*ls-tim;cry*^{02}) = 6, ZT_{max}(*s-tim;cry*^{02}) = 11.5. A Kruskal two-group comparison test was performed. p^*=0.017, p^{ns}=0.4. The lowest line of the box plot indicates the first interquartile, the centre line the median, the upper line the third interquartile, the cross the average, and the whiskers indicate the minimum and maximum except for out layers. **b, c)** TIM (blue) and PER (magenta) in the LNd (b) and the s-LNv (c) on day six of LLTC. PDF antibody (green) was used as a marker to identify the LN PDF^+ neurons. Scale bar: 10µm. Source data are provided as a Source Data file.

Supplementary Figure 5

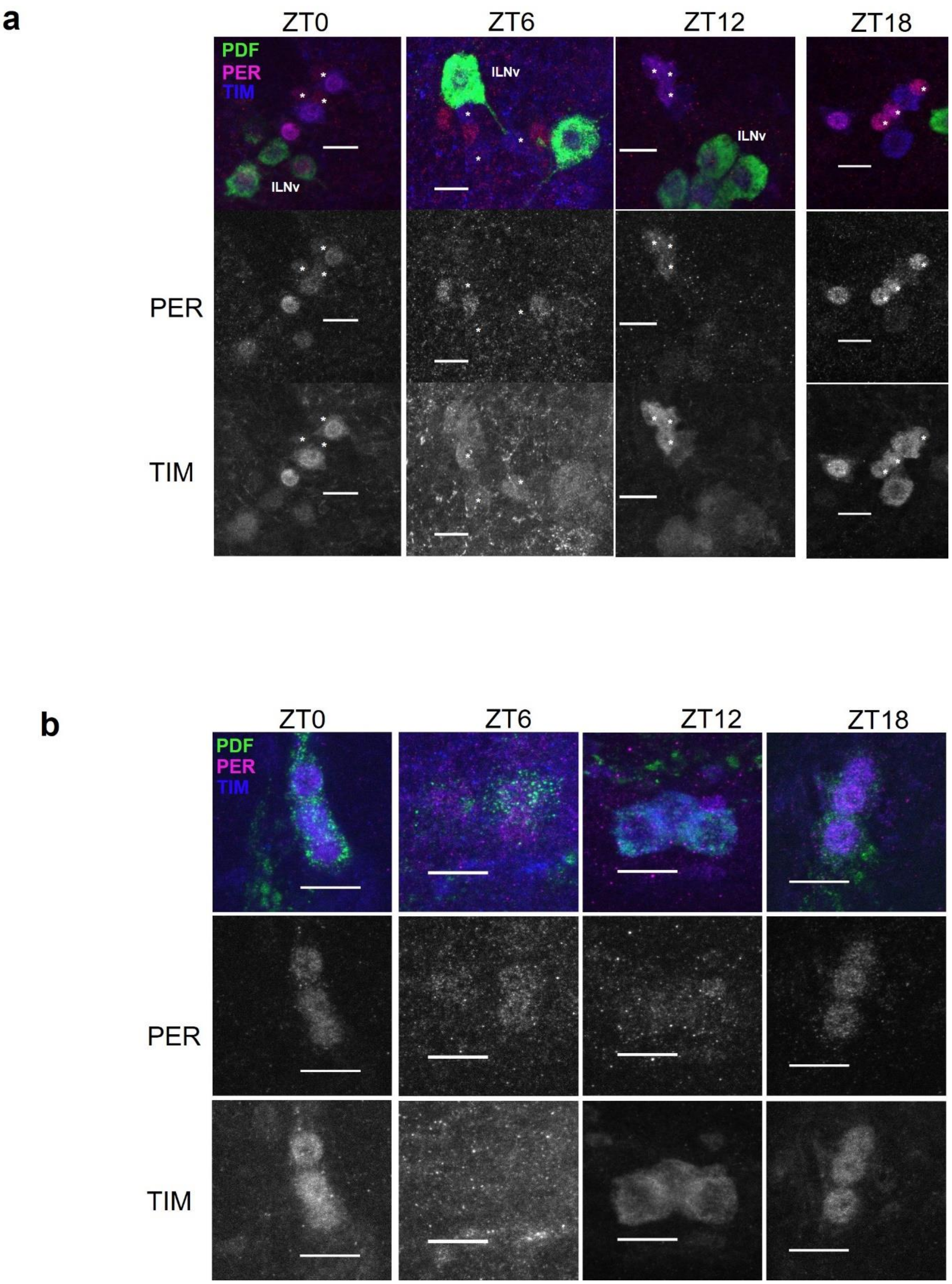

Supplementary Figure 5: PER and TIM staining in *norpA*^{P41} *cry*^{02} flies in *s-tim* background.

Representative images of quantification from Fig. 4c. TIM (blue) and PER (magenta) in the LNd **(a)** and the s-LNv **(b)** on day six of LLTC. PDF antibody (green) was used as a marker to identify the LN PDF^{+} neurons. The white stars in (a) label the LNd CRY^{-} Scale bar: 10µm.

Supplementary Figure 6

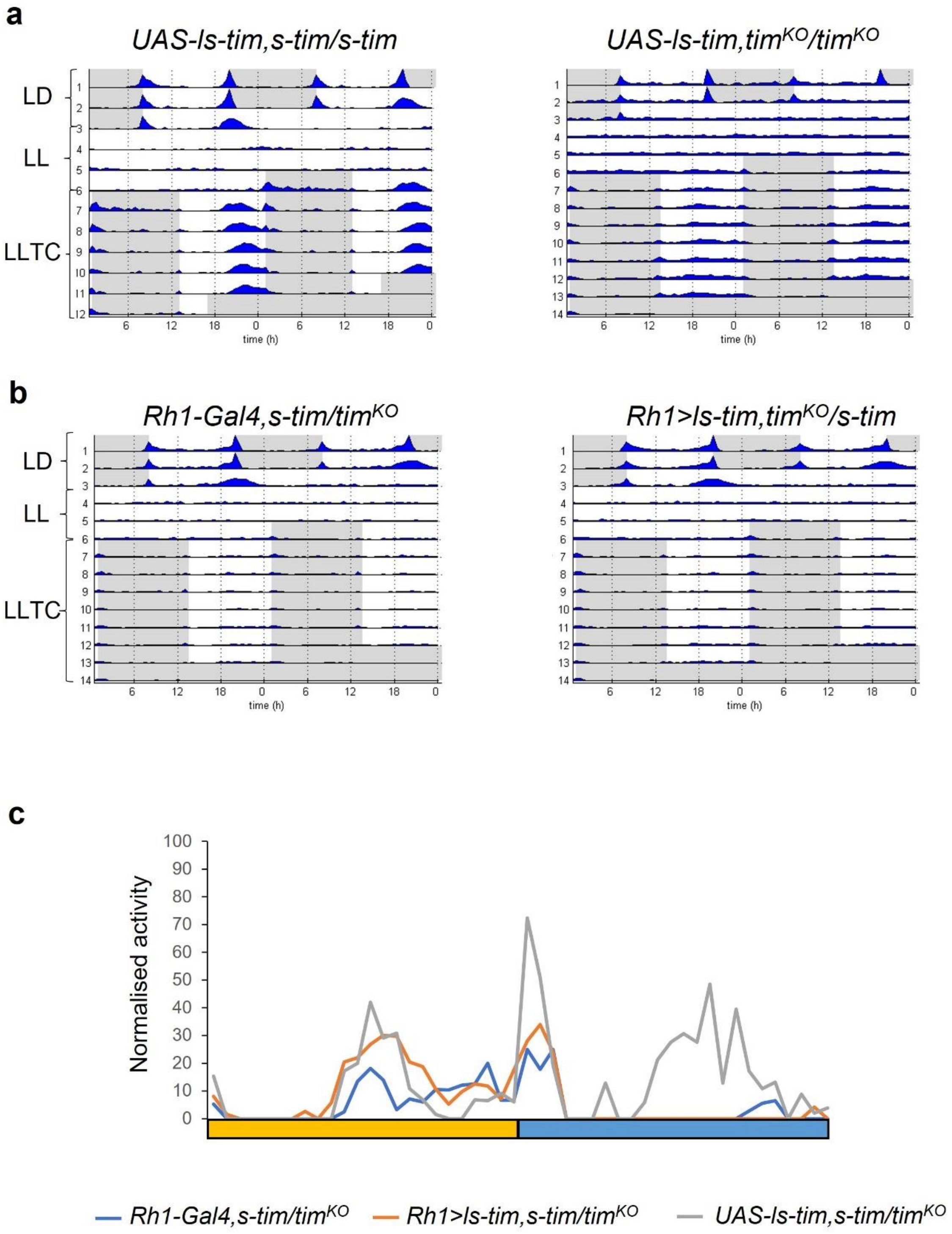

Supplementary Figure 6: Expression of *ls-tim* in photoreceptor cells is not sufficient to restore synchronisation to temperature cycles during constant light in *s-tim* flies. a) Group

actograms of control flies carrying the *UAS-ls-tim* construct in a homozygous *s-tim* background (N = 20) and *tim*^{KO} background (N = 17) as described in the legend to Fig. 1a. Note that the *UAS-ls-tim* flies in homozygous *s-tim* background synchronize to LLTC (upper left), indicating leaky expression of *UAS-ls-tim* in absence of a *Gal4* driver. However, the level of expression is not sufficient to restore synchronisation to LLTC (and LD) in a homozygous *tim*^{KO} background (upper right) or in heterozygous *tim*^{KO}*/s-tim* flies (Fig. 5a). **b)** Group actograms as in (a) for *Rh1-Gal4, s-tim/tim*^{KO} and *Rh1-Gal4, s-tim/UAS-ls-tim, s-tim, tim*^{KO}. N for both genotypes = 19. **c)** Median of normalised activity. Same flies as in b and Fig. 5. Source data are provided as a Source Data file.

Supplementary Figure 7

a

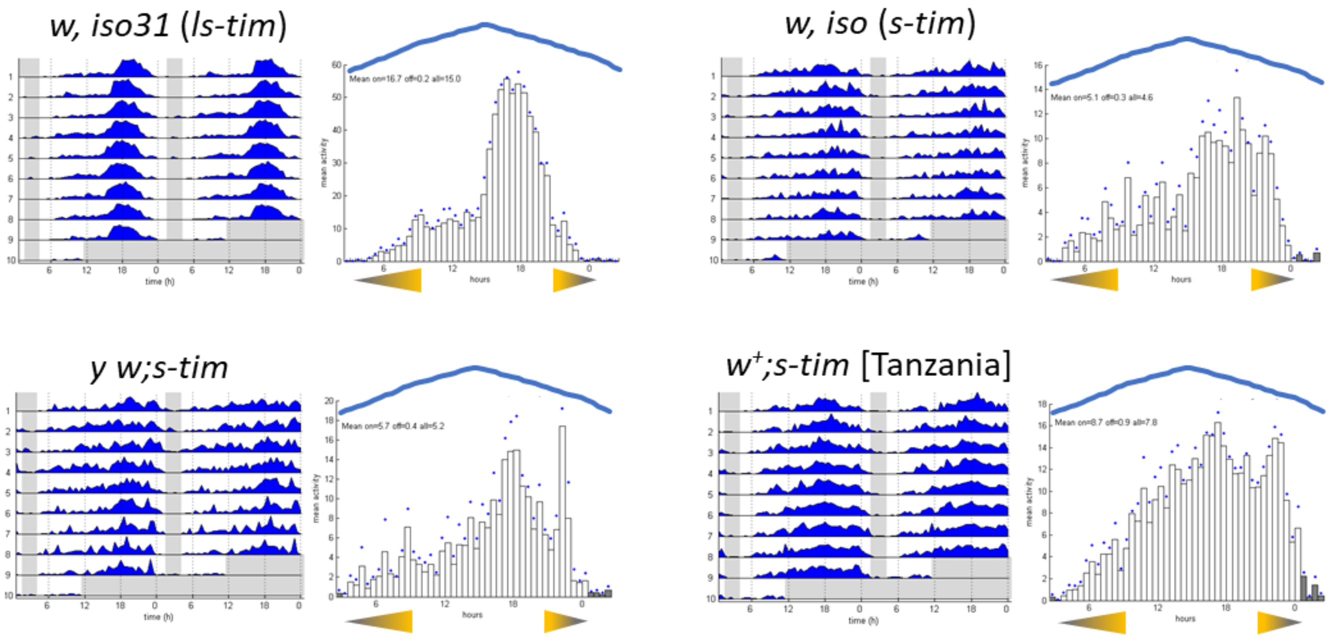

b

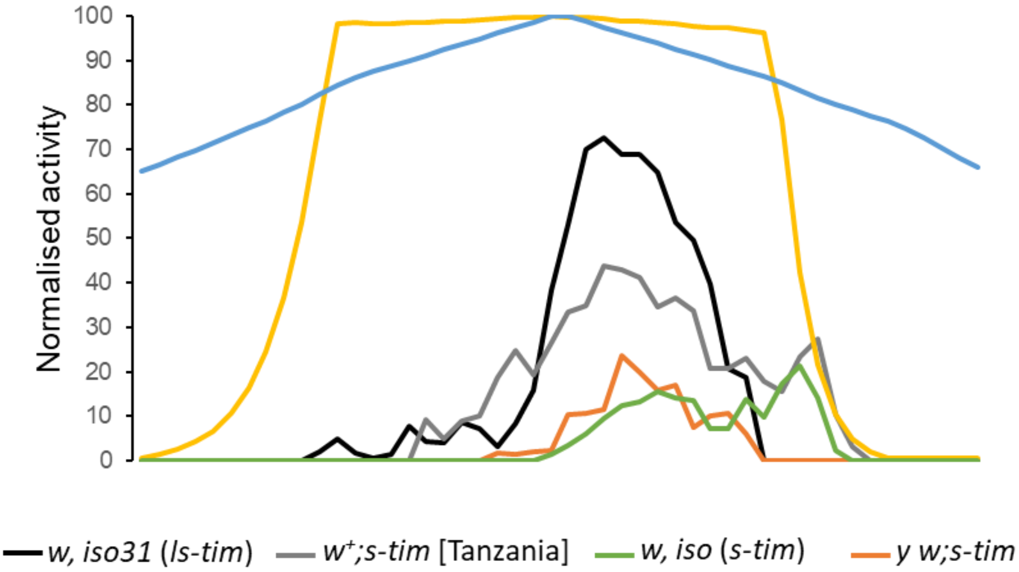

Supplementary Figure 7: Only *ls-tim* flies are able to synchronize to Northern latitude summer conditions. a) Group actograms and corresponding histograms of the last 3 days of one representative experiment as in legend to Fig. 6b. Blue diamonds indicate SEM. N (*w, iso31 ls-tim*): 20; (*w, iso s-tim*): 20; (*y w; s-tim*): 19; (w^+*; s-tim* [Tanzania]): 16. **b)** Median of the normalised activity of two experiments on the 6th day under Oulu conditions. For all the genotypes, apart from *s-tim* [Tanzania] (N=16), N=40. Source data are provided as a Source Data file.
