# Supplementary material for: A natural timeless polymorphism allowing circadian clock synchronization in “white nights”
Source: Nat Commun. 2022 Mar 31;13:1724. doi: 10.1038/s41467-022-29293-6 (PMC8971440; doi:10.1038/s41467-022-29293-6)
Supplement: Supplementary file 2 — Reporting Summary [file 41467_2022_29293_MOESM2_ESM.pdf]

nature portfolio

Corresponding author(s): Ralf Stanewsky

Last updated by author(s): Feb 25, 2022

Reporting Summary

Nature Portfolio wishes to improve the reproducibility of the work that we publish. This form provides structure for consistency and transparency in reporting. For further information on Nature Portfolio policies, see our Editorial Policies and the Editorial Policy Checklist.

Statistics

For all statistical analyses, confirm that the following items are present in the figure legend, table legend, main text, or Methods section.

n/a	Confirmed	
☐	☒	The exact sample size (*n*) for each experimental group/condition, given as a discrete number and unit of measurement
☐	☒	A statement on whether measurements were taken from distinct samples or whether the same sample was measured repeatedly
☐	☒	The statistical test(s) used AND whether they are one- or two-sided<br>*Only common tests should be described solely by name; describe more complex techniques in the Methods section.*
☒	☐	A description of all covariates tested
☐	☒	A description of any assumptions or corrections, such as tests of normality and adjustment for multiple comparisons
☐	☒	A full description of the statistical parameters including central tendency (e.g. means) or other basic estimates (e.g. regression coefficient) AND variation (e.g. standard deviation) or associated estimates of uncertainty (e.g. confidence intervals)
☐	☒	For null hypothesis testing, the test statistic (e.g. *F*, *t*, *r*) with confidence intervals, effect sizes, degrees of freedom and *P* value noted<br>*Give P values as exact values whenever suitable.*
☒	☐	For Bayesian analysis, information on the choice of priors and Markov chain Monte Carlo settings
☒	☐	For hierarchical and complex designs, identification of the appropriate level for tests and full reporting of outcomes
☒	☐	Estimates of effect sizes (e.g. Cohen's *d*, Pearson's *r*), indicating how they were calculated

Our web collection on statistics for biologists contains articles on many of the points above.

Software and code

Policy information about availability of computer code

Data collection	1) Behaviour: Drosophila Activity Monitoring System (DAM System). Commercially available at Trikinetics, Waltham, MA, USA: https://www.trikinetics.com/<br>2) Immunohistochemistry followed by confocal microscopy (Leica SP8)
Data analysis	1) Behavioural Analysis: MATLab fly toolbox: Levine et al (2002) BMC Neuroscience 3:1 and custom Excel Macro: Lamaze et al (2017) Sci Rep 7: 40304<br>2) Immunostaining quantification: ImageJ (NIH) (version 1.53n) as described in Lamaze et al (2017) Sci Rep 7: 40304<br>3) Estimation Statistics BETA (https://www.estimationstats.com/#/)

For manuscripts utilizing custom algorithms or software that are central to the research but not yet described in published literature, software must be made available to editors and reviewers. We strongly encourage code deposition in a community repository (e.g. GitHub). See the Nature Portfolio guidelines for submitting code & software for further information.

Data

Policy information about availability of data

All manuscripts must include a data availability statement. This statement should provide the following information, where applicable:

- Accession codes, unique identifiers, or web links for publicly available datasets
- A description of any restrictions on data availability
- For clinical datasets or third party data, please ensure that the statement adheres to our policy

Source data used to generate all plots and graphs are provided with this paper. The raw datasets generated consist of raw behavioural activity files and confocal microscope images. Due to the large size of these files they were not deposited in a public repository, but are available from the corresponding authors on

reasonable request. The data are saved on a SSD disk, on a computer and on the laboratory network.

Field-specific reporting

Please select the one below that is the best fit for your research. If you are not sure, read the appropriate sections before making your selection.

☒ Life sciences ☐ Behavioural & social sciences ☐ Ecological, evolutionary & environmental sciences

For a reference copy of the document with all sections, see nature.com/documents/nr-reporting-summary-flat.pdf

Life sciences study design

All studies must disclose on these points even when the disclosure is negative.

Sample size	according to the standards in the Drosophila circadian clock field (see e.g., Cichewicz and Hirsh 2018, Commun Biol 1, 25. doi.org/10.1038/s42003-018-0031-9
Data exclusions	No exclusions, unless indivduals died during the early stage of the experiment
Replication	Behavioural Experiments were replicated at least 3 times with flies collected from independent crosses, with similar results. Immunostainings were performed once per genotype and timepoint, but several brains were analyzed for each timepoint and genotype as indicated in the figure legends.
Randomization	n/a, because data were objectivly quantified as described in the manuscript
Blinding	n/a, because data were objectivly quantified as described in the manuscript

Reporting for specific materials, systems and methods

We require information from authors about some types of materials, experimental systems and methods used in many studies. Here, indicate whether each material, system or method listed is relevant to your study. If you are not sure if a list item applies to your research, read the appropriate section before selecting a response.

Materials & experimental systems

n/a	Involved in the study	
☐	☒	Antibodies
☒	☐	Eukaryotic cell lines
☒	☐	Palaeontology and archaeology
☐	☒	Animals and other organisms
☒	☐	Human research participants
☒	☐	Clinical data
☒	☐	Dual use research of concern

Methods

n/a	Involved in the study	
☒	☐	ChIP-seq
☒	☐	Flow cytometry
☒	☐	MRI-based neuroimaging

Antibodies

Antibodies used	1. Drosophila rabbit anti-Period (generated by R. Stanewsky, see Stanewsky et al 1997 below)<br>2. rat anti-Timeless (donated by Isaac Edery (see Sidote et al 1998, below).<br>3. Monoclonal anti-PDF C7: Hybridoma Bank: https://dshb.biology.uiowa.edu.<br>4. goat anti-mouse Alexa Fluor 488 (cross adsorbed) Invitrogen Catalog # A-32723<br>5. goat anti-rabbit Alexa Fluor 555 Invitrogen Catalog # A21428<br>6. goat anti-rat Alexa Fluor 647 Invitrogen Catalog # A21247
Validation	Stanewsky et al (1997), J Neurosci 17:676-696 (anti PER). Sidote et al (1998) Mol Cell Biol: 18:2004 (anti-TIM). Developmental Studies Hybridoma Bank: https://dshb.biology.uiowa.edu/ (anti-PDF C7)

Animals and other organisms

Policy information about studies involving animals; ARRIVE guidelines recommended for reporting animal research

Laboratory animals	Drosophila melanogaster (fruit flies) obtained from stock centers and other research labs as indicated in the manuscript
Wild animals	n/a
Field-collected samples	n/a

Ethics oversight	n/a

Note that full information on the approval of the study protocol must also be provided in the manuscript.
